# Motivating Pregnant and Breastfeeding Women in Spain to Avoid Persistent Toxic Substances in Their Diet

**DOI:** 10.3390/ijerph17238719

**Published:** 2020-11-24

**Authors:** Andres Fontalba-Navas, Eva Zafra Aparici, Maria Clara de Moraes Prata-Gaspar, Esther Herrera-Espejo, Miguel Company-Morales, Cristina Larrea-Killinger

**Affiliations:** 1 Antequera Hospital, Northern Málaga Integrated Healthcare Area, 29200 Antequera, Málaga, Spain; 2 Department of Public Health and Psychiatry, University of Málaga, 29016 Málaga, Spain; 3 Department of Anthropology, Philosophy and Social Work, University Rovira y Virgili, 43003 Tarragona, Spain; eva.zafra@urv.cat; 4 Department of Social Anthropology, University of Barcelona, 08007 Barcelona, Spain; ma_prata@hotmail.com (M.C.d.M.P.-G.); larrea@ub.edu (C.L.-K.); 5 Department of Psychology, University of Granada, 18010 Granada, Spain; estherpsicoree@gmail.com; 6 Seron Primary Care Center, Northern Almería Integrated Healthcare Area, 04600 Huercal-Overa, Almería, Spain; miguelcompanymorales@gmail.com; 7 Department of Nursing, Physiotherapy and Medicine, University of Almería, 04120 La Cañada, Almería, Spain

**Keywords:** pregnancy, breastfeeding, diet, persistent toxic substances, motivation, helplessness

## Abstract

The objective of this study was to explore what motivates pregnant and breastfeeding women to make changes in their diet, specifically to examine how their perceptions regarding diet facilitate or act as obstacles to introducing healthy eating habits. For the optimal development of the mother, the fetus, or breastfeeding baby, it is important to avoid foods containing substances, such as persistent toxic substances (PTSs), that are harmful to health during pregnancy and after the baby’s birth. This study used a qualitative research methodology, based on semi-structured individual interviews, food diaries, free lists, and focus groups with 111 pregnant and breastfeeding women in Spain. This approach was followed by a systematic and exhaustive exploitation of the qualitative data obtained, following the methodological principles of grounded theory. From the study results, we conclude that the motivation for a change in diet to avoid PTSs is based on the desire to promote good health, beliefs about the importance of having a varied diet, and the avoidance of potential risks. The main obstacles to change can be attributed to inadequate information, contradictory discourses, and socioeconomic difficulties.

## 1. Introduction

Food and diet have biological, social, cultural, and symbolic functions. Eating is a daily practice forming part of the construction of social identity [1,2,3]. The act of eating is complex, multidimensional, and forms part of reality, while also being socio-cultural and biological, determined both by socio-cultural and medical norms. Incorporating a food means—both in terms of the real and on the level of the imaginary—incorporating all of its properties. According to the “principle of incorporation” proposed by Fischler [1], in the biological sphere, the individual incorporates energy and nutritional properties; on the level of the imaginary, the symbolic, moral, and intellectual qualities of food are incorporated and finally, in the social sphere, incorporation generates collective identity and otherness.

In this way, given that human beings are omnivores and may find themselves specifically exposed to intoxication by food, and given that the incorporation of food is perceived as an act with important consequences, food and diet have always been an object of health concerns, in all societies. However, concern about the health-related aspect of food has intensified in recent decades [4,5]. The increase in life expectancy and the prevalence of chronic noncommunicable diseases, such as cardiovascular diseases, have brought new concerns regarding food, increasing the individual’s responsibility in relation to his or her own health and dietary practices [6]. In addition, processes of industrialization and urbanization, as well as technological advances, have disconnected individuals from the origin of the food they eat and the food chain. We no longer know exactly what we are eating, and therefore new risks arise, risks that have been reinforced by food crises (for example, the mad cow crisis) in recent decades [7,8]. According to Beck [9], with the industrialization of society, risks that can be invisible have become a constant in daily life in Western countries. This invisibility is particularly present in food consumption [10]. Modern food has become an “unidentified food object”, and industrialization and new agri-food production methods have led to a silent risk, related to the accumulation of synthetic chemical substances in the body [11,12] as is the case of persistent toxic substances (PTSs). Traditional diets that feature whole or minimally processed foods and emphasize home-cooking and food preparation are being replaced by diets comprised of industrially processed and prepared food products [13].

PTSs are human-made industrial substances, which cause concern and alarm due to their capacity to remain permanently in the environment, their capacity for long-range transport from primary emission sites, and their bio-magnification or bio-accumulation in ecosystems [14]. Their main characteristic is the danger they pose to the environment and human health [15]. Exposure can lead to chronic diseases, male reproductive problems, complications in pregnancy, certain cancers and obesity, and can affect brain development [16]. PTSs have a detrimental effect on the health of the mother and child, because they increases risk of fetal loss, intrauterine growth restriction, preterm birth, birth defects, respiratory and other childhood diseases, neuropsychological deficits, premature or delayed sexual maturation, and certain adult cancers linked to fetal or childhood exposures [17,18]. Internal pollution from the impact of these substances has important cultural, social, ideological, and economic implications [19,20]. In view of these considerations, the present study was undertaken to analyze the impact of PTSs on the lives of pregnant and breastfeeding women, as this is a period of the life cycle in which food is a central issue in women’s health.

Pregnancy and the breastfeeding period can provide a “learning moment” that leads to a change in diet toward salutogenic practices. The salutogenic model of health emphasizes assets for health, which include any factor or resource that enhances the capacity of individuals, communities, and populations to maintain health and well-being [21]. In the period of pregnancy and breastfeeding, women have greater concern for their health and are in regular contact with health professionals, such as obstetricians and midwives [22]. However, some studies have shown that among the changes taking place during pregnancy, there may be a decrease in physical activity [23]. Since overweight during pregnancy is a widely identified health problem, multiple strategies have been developed to standardize weight gain recommendations [24]. Despite these strategies, the motivation that can lead women to avoid the intake of chemical substances during pregnancy is a field that has yet to be explored in depth.

Given the lack of knowledge and the gap in the literature of the mechanisms influencing changes in behavior among pregnant and breastfeeding women, the main objective of our study was to explore their specific motivations for changing or maintaining eating practices, the methods used, and the factors influencing their quest for a healthy diet and physical activity. Detailed information on cognitive and psycho-social aspects of individual factors, such as motivation, which influence lifestyle behavior during pregnancy and in the postnatal period, would contribute to the development of informational strategies to advise pregnant women to avoid ingesting foods containing PTSs [25].

## 2. Materials and Methods

Although quantitative designs are commonly used in clinical research, some studies like this require qualitative methods [26]. The field work consisted of a qualitative study using semi-structured interviews with pregnant and breastfeeding women and health professionals, as well as the preparation of food diaries, free lists, and focus groups among the participants. Additionally included were four ethnographies in which the same techniques were applied, in addition to participant observation. The main contribution obtained from a semi-structured interview results from the density and level of complexity of the material obtained [27]. It is a method that seeks to understand how the individual views the social and the personal environments. This type of interview is relevant to the analysis of the meanings that social actors assign to their practices, and reveals the systems of values and norms on which these practices are grounded [28].

The food diaries created enabled us to record the eating practices of the pregnant and breastfeeding women who took part in the study. The focus groups, on the other hand, helped us promote interaction among the women, thus obtaining first-hand information. The women’s participation in these groups encouraged discussion and facilitated a flexible and open discourse, based on intersubjectivity and reflexivity. It also helped the researchers observe the women’s non-verbal behavior. The groups consisted of 10 women in the first group (six of whom were pregnant and four breastfeeding) and 8 in the second (four of whom were pregnant and four breastfeeding).

The above methodological instruments were complemented by field notes and in-depth observation during the four ethnographies, which enabled us to perform a triangulation between the different methods and the data obtained, to compare the preferences, norms, social representations, and food and health practices expressed in the different research contexts. This comparative approach also allowed us to observe how medical and nutritional norms and recommendations are carried out, modified, or ignored by the pregnant and breastfeeding women in our study groups [29,30].

### 2.1. Sample, Participants, and Context

Both the health centers and the pregnant and breastfeeding women included in this research were selected using an intentional or rational non-probabilistic sample. A fair and non-discriminating selection of the sample was made, seeking the elements that best represented the object of study, not simply those which were most accessible. The inclusion criteria applied were that the women must have been born in Spain, that the pregnant women should be at 20 weeks of gestation, that the women who were breastfeeding (maternal and/or artificial) should have been doing so for no more than six months, and that the women should represent a wide range of socioeconomic strata.

The field work was carried out at eight healthcare centers (three hospitals and five primary healthcare centers) in two Spanish regions, Catalonia (Barcelona and its metropolitan area, Baix Llobregat, Tarragona, and Ribera d’Ebre) and Andalusia (Granada and its metropolitan area and Valle del Almanzora in Almería). The pregnant and breastfeeding women were recruited via these hospitals and primary healthcare centers.

The interviews and the application of the food diaries began in January 2016, following approval for the study from the corresponding ethics committees, and ended in September of the same year. All participants were informed of the study goals and methods. All procedures performed in studies involving human participants were in accordance with the ethical standards of the institutional and/or national research committee and with the 1964 Helsinki Declaration and its later amendments or comparable ethical standards. This study was approved by the Ethical Committee of Clinical Investigation of Almería code CONRESMUM 08-2016. Informed consent was obtained from all individual participants included in the study.

To address the study goal of determining motivations for changing eating habits, the following issues were explored:

1. Advice on dietary changes or continuity.
2. Foods that the participants believed contained chemical substances, inquiring about the dangers and trust associated with these foods and the women’s reasons for such beliefs. When necessary, the topics of pesticides, preservatives, metals, sweeteners, etc. were discussed.
3. Participants’ perceptions regarding the long-term accumulation of these chemical substances in the body and how they are eliminated and transmitted.
4. Knowledge about PTS and verification in the discourse of the participants. Inquiry into their knowledge in this respect and whether they employed practices or strategies to avoid them in everyday life.

### 2.2. Data Categorization and Analysis

The discourses of the pregnant and breastfeeding women were recorded in digital audio format during the interviews and focus group sessions. In addition, during these sessions, two of the researchers took notes and observed the non-verbal language of the participants while another researcher moderated the meeting. During the ethnographic work, the ethnographers took field notes that were later digitized for computer analysis. Similarly, the food diaries and the free lists were transformed into digital format.

The transcripts of the interviews and focus group sessions were made by a specialist service provider. The Atlas-Ti 7 computer program was used to encode the content of the discourses thus obtained.

Following the procedure for qualitative research, our analysis revealed the essential content of the discourses obtained, described the relationships between these discourses, and compiled the data into an organized whole. As part of the coding process, the information obtained was synthesized and categorized appropriately, according to the discourse topics addressed (Table 1).

After the content analysis, an interpretive (hermeneutical) analysis of the discourse was performed. This broad overview of the study findings enabled us to relate the values and beliefs underlying the women’s discourses with various social theories.

## 3. Results

**Figure 1:**
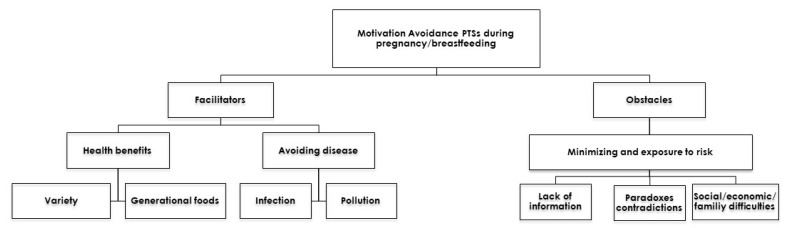
Conceptual map of factors that increase or decrease the motivation for avoiding Persistent Toxic Substances in the diet during pregnancy/breastfeeding.

In attempting to understand the factors that motivate or obstruct dietary changes by pregnant and breastfeeding women, we started from the principle that obstacles and facilitators can be defined as those factors that prevent or facilitate, totally or partially, the implementation of a change [31]. Given that eating is a plural act, the result of different determinants, we believe that these obstacles and facilitators are associated with different dimensions: biological, social, cultural, psychological, symbolic, material, etc. The discourse analysis performed enabled us to construct a conceptual map (Figure 1) separating the aspects that motivated the women to make a dietary change from the obstacles to doing so.

The following categories and sub-categories are included in the analysis.

### 3.1. Motivation for Dietary Changes during Pregnancy/Breastfeeding

#### 3.1.1. Rewards—Promoting Health

According to the discourses analyzed, a key factor that leads women to change their dietary practices is concern about their own and their child’s health. Within this category, we identify two sub-categories associated with perceptions about health-giving nutrition: the need to have a varied diet and an idealized view of the diet of the past, two notions that are discussed below.

##### Variety

From the perspective of nutritional science, the concept of a varied diet refers to incorporating a great diversity of foods into the diet in order to maintain an adequate energy balance [32]. The World Health Organization’s general and global recommendations for a healthy diet [33] have been incorporated into dietary guidelines in many countries—including the Naos Strategy in Spain [34]—as the standard for an adequate diet to increase energy consumption and to provide a more diverse contribution of foods and nutrients. This dimension appears in the discourse of the participants, who emphasize the desirability and necessity of a varied diet. Virginia (pregnant, 40 years old, Barcelona) talks about a greater concern for having a varied diet to guarantee a supply of adequate nutrients during pregnancy:

“I guess you have to pay more attention to the vitamins you get, you know? You need to take vitamins, but especially, have more fruit and vegetables, raw ones, to get all the nutrients. This is a little bit of what I would vary from what I usually eat”.

(Virginia, pregnant, 40, Barcelona)

Another idea that appears, often associated with the idea of variety, is the expression “eat a little bit of everything,” as expressed in the following discourse:

“A little bit of everything, you know? You need to eat everything like…vegetables, fish, meat, eggs, a variety… I mean, a varied diet”.

(Luisa, breastfeeding, 28, Granada)

The idea that a varied diet is a healthy diet is not only found among pregnant and breastfeeding women. In fact, several studies conducted with different social groups from different countries revealed that variety is often among the factors used to describe healthy eating [35,36,37,38,39]. The University of Barcelona Food Observatory found that there is a consensus among the Spanish population that to “eat healthy” you have to have a varied diet, a little bit of everything and balanced. According to the Eurobarometer on food and health, Spain is the second country (after Belgium) where eating a varied and balanced diet is the idea most cited by individuals (75%) to define a healthy diet.

The results of this study indicate that pregnant and breastfeeding women emphasize this dietary norm of eating a variety of foods daily.

##### Generational Foods: From Mothers to Grandmothers 

Certain foods and/or ways of preparing foods that are considered “traditional” are one of the elements differentiating the food models of each country and culture. These foods and/or preparations are an important part of the culture, history, identity, heritage, and local economy of a place, region, or country. They are commonly perceived as foods that have been consumed locally or regionally for a long time, and the methods of preparation for consuming them have been passed on from generation to generation [40]. In this regard, based on our discourse analysis, various participants express their trust in the food and recipes of their ancestors, especially those handed down from their mothers and grandmothers. The emotional and family dimension of food is therefore a determinant of the choices and eating behavior of pregnant and breastfeeding women. Their trust in women from previous generations is also directly related to those women having had children and often grandchildren and having taken care of them, thus the empirical experience in the life of each of our informants transforms into a model.

“The other day someone told me that we should go to the supermarket imagining we were our great-grandmothers, and buy what they would buy. I suppose nothing would have been in a bag or package or frozen. And it seems to me that’s what it’s about, that the right thing and the good thing is that. I should stop thinking about what my great grandmother would have bought”.

(Susana, breastfeeding, 40, Barcelona)

In fact, we find an idealization of the diet of the past, an idealization that is often associated with the image of older people, especially grandmothers. The women in our study connect the food of the past to “tradition”, to products perceived to be natural, local, and less industrialized and processed, to a type of consumption that was not part of fast food culture and eating out but in the domestic sphere. Such representations are also found among individuals from different industrialized western countries [41].

The social transformations that have occurred in the last 100 years have affected the temporal and spatial dynamics of eating. As mentioned earlier, industrialization and urbanization have disconnected individuals from the origin of products, and technological advances increasingly produce foods that are “unknown” and unidentifiable by consumers. This disconnection exacerbates the anxiety involved in the incorporation of foods [5] and in a society in which everything becomes uncertain, the past is valued as an ideal. In fact, if representations of the past structure individual and group identities in food modernity [39], the desire to return to the past signifies regaining security, one’s identity, and belonging to a group threatened by social transformation. As Poulain suggests [6], the interest of individuals in local food today, the “terroir” invokes nostalgia for a “social space” in which they lived “sheltered by an identifiable and identifying food culture”.

#### 3.1.2. Avoiding Harm or Illness

While changes in diet are motivated by the quest for better health, according to the informants’ discourses, they may also be associated with a desire to avoid harm and illness. In fact, these are two complementary and interrelated aspects. As in the case of the desire to be healthy, it is not a change focused only on oneself but also on the baby. These mothers, from the first moments of their children’s live, take responsibility for what they ingest, developing strategies based on different information and experiences. For example, this pregnant woman explains the responsibility she feels regarding the health of her baby:

“When you aren’t pregnant, you’re just on your own, so it’s a little like, if one week you’re a little more relaxed, and you eat an ice cream every day, it doesn’t matter, or if you have a Coke or whatever. I feel like, well, it’s my body, and there’s no problem; it doesn’t matter. But, when you have a baby inside you, it’s like what you’re eating, you pass on to the baby, so I think about it. If I drink a coke, it’s like I’m pouring coke into the baby, and I don’t know, it’s different”.

(Barbara, pregnant, 36, Barcelona)

In another study, health professionals who had strongly internalized the concepts of mother-to-child transmission and the accumulation of chemical substances acknowledged a potential health risk in some cases [42]. Two sub-categories within this category, infection and pollution, can be identified, and these topics are discussed below.

##### Infection 

The prevention of infectious diseases during pregnancy—given their potential for teratogenic damage, as in the case of toxoplasmosis [43]—forms part of various preventive health campaigns that have been carried out for many years in Spain, identifying cases that are at risk and making recommendations for this population. As a result, a preventive discourse has been acquired and incorporated as a fundamental part of food and diet during pregnancy. One of our study participants expressed this as follows:

“Well, since I haven’t had toxoplasmosis, right, well, of course, for example, I can’t eat chorizo, ham, or sausage, and so normally, I always take a sandwich to work, and normally, it’s either sausage or ham or something. So now we’ve changed; I have tuna, an omelette or … something like that”.

(Isabel, pregnant, 36, Barcelona)

##### Pollution

Exposure to pollutants during pregnancy and early life can have adverse health effects [44]. Consideration is given to outdoor air pollution, water pollution, allergens and biological organisms, metals, pesticides, primary and secondary smoke, persistent toxic compounds, noise, radiation, and occupational exposures. In the discourses analyzed, exposure to pollutants was identified and related to direct health consequences:

“They accumulate in the body. You can’t eliminate heavy metals. And fish carry heavy metals, the very large ones. And your body doesn’t eliminate them, like lead, mercury; it all stays there. And pesticides, I don’t know what to say. It’s that, um, often I think that more than them accumulating, it’s the alterations they cause internally. Because, there are also free radicals, how to fight them, all that promotes aging. And externally, and also with the organs. I mean, I no longer think it’s a matter of accumulation, but it’s about genetic alteration”.

(Teresa, pregnant, 38, Macael, Almería)

### 3.2. Obstacles to Change

Obstacles are defined as difficulties in adapting to transformations that take place in one’s environment, both internally and externally, even more so if the change is difficult or costly. Change is the variation or passage from a previous situation to another desired situation, based on a vision [45]. In the case of food and diet during pregnancy and breastfeeding, we identified the following obstacles to changing dietary habits to eliminate contaminants in the diet:

#### 3.2.1. Need for Better Information 

Despite the evidence described above regarding exposure to PTS, the discourses also reflect a lack of knowledge and a lack of adequate information on the subject. Since the risk is not understood by the informants, it is difficult to make recommendations on what steps should be taken to avoid PTS. This health innumeracy about PTS might also affect health care professionals, who are often poorly prepared to explain food safety issues and recommendations to patients [46]. A previous study also found problems with ignorance about these compounds in the diet [47]. Lack of information is one of the most important points to keep in mind regarding subsequent intervention, as having more information on the subject would facilitate subsequent decision-making:

“Well, yes, it sounds familiar, the whole topic of pesticides commented on (…) I think everything has … but well, there are many more bad things that cause cancer, or diabetes because what can you eat? Everything seems to cause something”.

(Laura, pregnant, 36, Barcelona)

These observations are consistent with the findings drawn from our own study of health professionals, which revealed that they are aware of the information overload that women receive during pregnancy and lactation, as well as the responsibility women feel they have, which is related to the gender role that society assigns them.

#### 3.2.2. Paradoxes and Contradictions in the Discourses

Numerous contradictions place people in a difficult situation when choosing “what and how to eat” in a food social space such as ours. Characterized by unprecedented abundance, but also with different and often contradictory messages and discourses, we are subject to a food cacophony [1].

In this regard, several of the women in the present study emphasized the conflicts they often encounter in choosing the most appropriate diet for their baby during pregnancy. These conflicts are at the crossroads of multiple normative discourses: the anxiety vis-à-vis unknown food products, the agri-food industry, their personal expectations in trying to consume a healthy diet, and their level of knowledge.

“I almost don’t trust anything because it’s all about money, so everything is super manipulated, super bought off. I don’t believe anything; they tell me that something is super healthy, and a year from now they tell me that it wasn’t so healthy”.

(Cristina, 39, breastfeeding, Tarragona)

“I think I could probably do better; I could take better care of myself, not eating so many sweets, for sure, as in all areas of life, of course, but I’m relaxed. In the end, if you eat something with pleasure, it can’t be that bad for you!”.

(Raquel, 35, breastfeeding, Tarragona)

#### 3.2.3. Social, Economic, and Family Difficulties in Having a Healthy Diet without PTS 

Although knowledge and information are key aspects in determining food choices and practices, this study has shown that even with information on the toxic substances that foods may contain, many women feel pressure to disregard this information because of their circumstances or social/family situations. The difficulty in balancing work and family life, the higher prices of organic products, and less accessibility to them are some of the factors conditioning the food choices of the women interviewed:

“I understand that local products would be more ideal, but sometimes the prices are just too high; if you buy for a single person … but if you’re buying for a whole family, local products seem very expensive to me (…) And, besides, I don’t have time to go to the main market today, to the neighborhood street market on Saturday, etc. If you have time, it’s great, but that’s not my case. In the end, you have to choose…”

(Yolanda, 33, breastfeeding, Tarragona)

The adoption of behavior perceived to be healthier may be an individual decision, based on information or medical guidance [48], but value systems, cultural references, and the material and structural limitations of everyday life must also be considered. In fact, whether information is accepted or rejected and how it is interpreted depends on various factors, among them, the beliefs and social representations of each individual and socio-cultural group

## 4. Discussions

Studies in the field of food and health have shown that important discrepancies can exist between the norms and knowledge that individuals are aware of and their actual practices [32]. These studies highlight the complexity of the act of eating, which is the result not only of social, cultural, symbolic, and physiological factors but also of material ones associated with the temporal, structural, and financial issues of daily life. Thus, food practices may be modified according to the time of the week, the social environment, the individual’s responsibility vis-à-vis the family or work, etc. Poulain states that:

“Trying to change eating habits through information without first questioning the origin of these habits rests on several assumptions that are not true, such as: (a) that individuals are quite fixed in their erroneous practices; (b) the social environment is more or less stable; (c) nutritional knowledge is definitive”

[49]

In the same line, reference [3] point out that a change in nutritional knowledge does not necessarily lead to a change in dietary practices. We may have a lot of knowledge about nutrition and food, but social circumstances may mean we do not follow this understanding. In other words, information alone is not sufficient motivation for changing what we eat.

According to the ecological theory of behavioral change, diverse factors intervene in behavior, which can be classified in three levels [50]:

- Contextual: the sphere in which daily life takes place.
- Personal: genes, beliefs, abilities, attitudes.
- Social: family relationships, friendships, and relationship to the community in general.

Changing a behavior requires that the changes be maintained for a long period of time. In addition, changes will not be effective if they are only focused on one of the levels mentioned. To be long-lasting, changes must impact on all three levels simultaneously. 

In recent years, various new models of behavioral change have emerged in the field of psychology, which include emotional influences, habits, routines, and economic factors. 

These considerations are consistent with the theory of self-determination, in which internal motivation is the most powerful element. García del Castillo et al. developed their own theoretical construction based on this model, including the perception of risk and vulnerability (helplessness) as elements mediating in intrinsic (self-determination) and extrinsic motivation, and even having some effect on demotivation [51]. One of the theoretical possibilities addressed was that an increased perception of risk and vulnerability may heighten the probability of reducing demotivation, in which case the imposition of external regulation would become less necessary [52]. These ideas underscore the vital importance of mothers having access to information, fueling the knowledge and beliefs that will stimulate their internal motivation. Nevertheless, according to the self-determination model, although internal motivation is more important than external motivation, it cannot be denied that external influences also exert a significant impact [53].

The motivation to make any change is related to the activity of the brain reward circuit and to being able to imagine the future benefits that might be gained from achieving a certain objective or from avoiding potential harm. In our context, changes will only take place when the pregnant/breastfeeding woman perceives that she has control to make the necessary adjustments and that the results will be meaningful. Therefore, with regard to dietary habits, the motivation of pregnant women is closely related to learned helplessness, a situation in which the subject perceives that she does not have the power to make the changes needed to improve her situation. With most of the interviewees, this phenomenon is manifested in the repeated statement “we don’t know exactly what we eat” or “we have no choice but to buy what’s in the supermarket”.

Overcoming feelings of helplessness in relation to consuming a ‘healthy’ diet requires a cognitive effort to modify passive behavior related to eating habits; in this situation, both intrinsic and extrinsic motivation are important. Extrinsic motivation depends on external factors, such as the availability of foods, their cost, and the possibilities of the family budget. However, it is intrinsic motivation that brings about the greatest change and causes it to become well-established

Improving access to factual information should be accompanied by mechanisms to standardize and facilitate the comparison of the daily discourses on food we receive from different health, scientific, historical, environmental, industrial, and commercial spheres. In addition, conditions for economic and socially equal access to healthy toxin-free foods, such as organic foods, should be promoted, given that socioeconomic difficulties are important obstacles to pregnant and breastfeeding women changing their diets. As observed above, the modification of nutritional knowledge does not necessarily imply a modification of dietary practices, as a person or group may follow or disregard recommended dietary guidelines depending on their social circumstances [54]. Therefore, information alone does not guarantee a change in eating behavior. In short, information is more effective if it is accompanied by strategies that also impact on the social and economic context in which an individual lives.

To be effective, a campaign seeking to reduce the consumption of PTSs during pregnancy and breastfeeding must address intrinsic, personal, and health-related factors, in addition to focusing on the potential damage of PTSs. Furthermore, such an intervention should be collaborative and transversal, involving various public entities, to offer a creative and salutogenic model of health promotion being able to include other health habits, such as physical activity, which are very profitable in this period [55]. The proposed strategy for these women may be health education; it is to be aware of the sources of exposure and avoid them as much as possible. It has been shown that we can significantly reduce exposure by following a healthy lifestyle and the adoption of certain changes in eating habits.

As in the case of efforts to promote breastfeeding, our study results emphasize the importance of applying communication skills focused on individuals and relationships in order to encourage women to avoid the PTSs present in foods [56]. The kind of communication necessary must build trust, showing empathy and listening and responding to the needs of women. Organizational systems and services that facilitate continuity in care and time spent with women (for example, the continuity of care, before and after childbirth, or the availability of peer support models) are factors that will facilitate change [57]. Health professionals should be well apprised of social support in these circumstances in order to better assess the needs of pregnant and breastfeeding women [58]. This would make it possible to develop a comprehensive communication plan to help them avoid foods with a risk of PTS contamination. Such a plan should address food production, distribution, preparation, and consumption, among other areas, and in every case take into account the socio-cultural characteristics that determine these factors.

## 5. Conclusions

The motivation for a change in diet to avoid PTSs is based on the desire to promote good health, beliefs about the importance of having a varied diet, and the avoidance of potential risks. The main obstacles to change can be attributed to inadequate information, contradictory discourses, and socioeconomic difficulties.

## Figures and Tables

**Table 1:** Characteristics of the pregnant and breastfeeding women interviewed, 2016.

Pregnant and Breastfeeding Women				
Profiles	Pregnant	Breastfeeding	Total	
	62	49	111	
Age range	Age—20–29	Age—30–39	Age—40+	
	17	81	13	
Education level	Primary	Secondary	Higher	
	6	37	68	
Number of children	Child-1	Child-2	Child-3 or +	
	58	45	8	
Province	Almería	Barcelona	Granada	Tarragona
	30	51	20	10

(Source: The authors).
